# Predictors of physical and mental health-related quality of life outcomes among myocardial infarction patients

**DOI:** 10.1186/1471-2261-13-69

**Published:** 2013-09-10

**Authors:** Anna L Hawkes, Tania A Patrao, Robert Ware, John J Atherton, Craig B Taylor, Brian F Oldenburg

**Affiliations:** 1 School of Public Health and Social Work, Queensland University of Technology, Kelvin Grove, Brisbane, Australia; 2 School of Public Health, Tropical Medicine and Rehabilitation Sciences, James Cook University, Townsville, Australia; 3 Menzies School of Health Research, Brisbane, Australia; 4 School of Population Health, The University of Queensland, Herston, Brisbane, Australia; 5 Department of Cardiology, Royal Brisbane and Women’s Hospital and University of Queensland School of Medicine, Herston, Brisbane, Australia; 6 Department of Psychiatry, Stanford Medical Center, Stanford, California, USA; 7 Department of Epidemiology and Preventative Medicine, Monash University, Melbourne, Australia

**Keywords:** Myocardial infarction, Secondary prevention, Cardiac rehabilitation, Telephone-delivered, Health-related quality of life, Health coaching, Tele-health

## Abstract

**Background:**

Health-related quality of life (HRQoL) is an important outcome for patients diagnosed with coronary heart disease. This report describes predictors of physical and mental HRQoL at six months post-hospitalisation for myocardial infarction.

**Methods:**

Participants were myocardial infarction patients (n=430) admitted to two tertiary referral centres in Brisbane, Australia who completed a six month coronary heart disease secondary prevention trial (ProActive Heart). Outcome variables were HRQoL (Short Form-36) at six months, including a physical and mental summary score. Baseline predictors included demographics and clinical variables, health behaviours, and psychosocial variables. Stepwise forward multiple linear regression analyses were used to identify significant independent predictors of six month HRQoL.

**Results:**

Physical HRQoL was lower in participants who: were older (*p*<0.001); were unemployed (*p*=0.03); had lower baseline physical and mental HRQoL scores (*p*<0.001); had lower confidence levels in meeting sufficient physical activity recommendations (*p*<0.001); had no intention to be physically active in the next six months (*p*<0.001); and were more sedentary (*p*=0.001). Mental HRQoL was lower in participants who: were younger (*p*=0.01); had lower baseline mental HRQoL (*p*<0.001); were more sedentary (*p*=0.01) were depressed (*p*<0.001); and had lower social support (*p*=0.001).

**Conclusions:**

This study has clinical implications as identification of indicators of lower physical and mental HRQoL outcomes for myocardial infarction patients allows for targeted counselling or coronary heart disease secondary prevention efforts.

**Trial registration:**

Australian Clinical Trials Registry, Australian New Zealand Clinical Trials Registry, CTRN12607000595415.

## Background

Coronary heart disease (CHD) affects many people worldwide and life expectancy continues to improve after medical treatment [1]. Consequently there is particular interest in identifying characteristics associated with impairments or improvements in the quality of these extended life years [2]. Advances in treatment have left practitioners with numerous treatment alternatives offering no clear survival benefits at substantial cost [3], and so health-related quality of life (HRQoL), a measure of perceived well-being and ability to function physically, mentally, socially and emotionally, is increasingly being used as an outcome measure in trials designed to evaluate the quality of care for myocardial infarction (MI) patients [4,5].

The growth in interest in HRQoL outcomes has paralleled the increasing recognition of the importance of a patient’s perspective of his or her health status after medical treatment. Hence, HRQoL outcomes can play a role in the clinical management of patients by providing an additional and complementary measure to objective biomedical outcomes [6]. For MI patients, researchers have reported significant negative effects of CHD on HRQoL. In turn, poor pre-hospitalisation HRQoL and low scores in physical functioning have been associated with poorer post-hospitalisation general health, higher readmission rates, and increased mortality [7].

Studies investigating HRQoL after MI have identified several non-cardiac characteristics as the strongest predictors of HRQoL such as age [8-10], sex [10,11], education, non-cardiac co-morbidities [9], diet [12], depression [9,10], anxiety [13], and baseline HRQoL scores [14]. However, these studies have suffered from a range of limitations including: small sample sizes; select populations (primarily white, middle-aged men or patients receiving specific interventions); relatively short follow-up; limited baseline information on relevant patient characteristics that could contribute to HRQoL; and non-standard measurement of HRQoL [2,8].

Given the importance of HRQoL as an outcome, there is a need to identify predictors of lower HRQoL in a representative sample of patients using a widely used, valid and reliable outcome measure. It is important to collect information on a wide range of potential predictors including demographic and clinical variables, as well as behavioural and psychosocial characteristics as these are known to impact on long term prognosis [15]. Identification of these predictors would allow physicians to determine at the time of admission those patients who were more likely to report lower HRQoL, thus permitting appropriate risk stratification and management [16]. In addition, identification of these predictors would allow for appropriate multivariate adjustments when comparing HRQoL outcomes among MI patients receiving different treatments [14].

For this study, we used data from a randomized controlled trial of a telephone-delivered CHD secondary prevention program (ProActive Heart) in a sample of MI patients from across the state of Queensland, Australia [17,18]. HRQoL was assessed with the Short Form-36 (SF-36) [19]. The aim of this report was to identify demographic and clinical, behavioural, and psychosocial predictors of HRQoL for MI patients at six months post-hospitalisation.

## Methods

### Setting and participants

This study was conducted among participants with newly diagnosed MI between December 2007 and March 2010. Participants were from two hospitals in Brisbane, Australia (Royal Brisbane and Women’s Hospital and The Prince Charles Hospital). Data were collected as part of a randomised controlled trial (ProActive Heart) of a six month personalised telephone-delivered health coaching intervention (10 × 30 minute sessions) after discharge from hospital compared with usual medical care. The methods of the ProActive Heart trial have been described in detail previously [18]. Ethical approval was obtained from The Prince Charles Human Research Ethics Committee (EC2738), The Royal Brisbane and Women’s Hospital Human Research Ethics Committee (2007/049) and Monash University Human Research Ethics Committee (2007/0584MC), and written informed consent was obtained from each participant.

### Measurement

Assessments were completed at baseline (1–2 weeks after hospitalisation for MI) and at 6 months post-hospitalisation. Baseline data were collected from hospital medical records (demographic and clinical variables) or by trained computer-assisted telephone interviewers who were blind to study condition (behavioural and psychosocial variables). Six month data were collected by computer-assisted telephone interview.

### Outcome variables

The SF-36 Health Survey Version 2 [20] was used to measure HRQoL. It demonstrates good reliability and validity within cardiac populations [21], and population norms are available. It includes 36 questions yielding an 8 scale profile of functional health [physical functioning, role-physical, bodily pain, general health, vitality, social functioning, role-emotional and mental health as well as 2 psychometrically based physical (physical component summary score) and mental HRQoL (mental component summary score) summary scores] [20]. Available normative population data from the Medical Outcomes Study in the United States of America showed that participants with a physician-reported MI within the previous year had a mean (SD) physical HRQoL score of 42.7 (10.0) and mental HRQoL score of 51.7 (8.2) for people aged 55–64 years [22].

### Predictor variables

#### Demographic and clinical variables

Baseline demographic and clinical variables included gender, age, marital status, education, income, CHD medical procedure, co-morbidities (diabetes, hypertension), BMI [kg/m2; normal weight (≤25 kg/m2), overweight/obese (>25 kg/m2)] and waist circumference (cm; participants were sent a tape measure at all assessment points). Group assignment (usual care, intervention) was also included as a predictor variable.

#### Health behaviours

Health behaviours included self-reported: physical activity, physical activity self-efficacy and intention, television (TV) viewing, diet (fruit, vegetables, total fat, saturated fat, sodium, dietary cholesterol), alcohol intake (standard drinks per day) and smoking (yes/no).

Physical activity was measured using the Active Australia Survey [23] which is reliable and has acceptable validity within the Australian population [24], and test-retest reliability of the survey is similar to the International Physical Activity Questionnaire [25]. Weekly physical activity was calculated by adding together the time spent walking, in other moderate-intensity physical activity, and in vigorous intensity physical activity (vigorous activity was double weighted to account for additional energy expenditure). Physical activity was classified as sufficiently active (yes/no; ≥150 minutes moderate to vigorous physical activity/week) [15]. Two measures were used to measure physical activity stage of change and self-efficacy. A 5-item measure of physical activity intention was used to determine participants’ stage of change (pre-contemplation, contemplation, preparation, action or maintenance). A 10-point scale was used to rate level of confidence (self-efficacy) in exercising over the next 6 months with one indicating “not at all confident” and 10 ”very confident” [26]. TV viewing time was collected as an estimate of the total time spent watching TV, on an average day, over the past month. Self-reported TV viewing has been shown to be a reasonably reliable and valid measure of sedentary behaviour for adults [27].

Diet was assessed with a validated food frequency questionnaire [28] that estimated intake of most nutrients accurately (within 10%) and did not systematically under- or over-estimate against weighted records. Alcohol was categorised based on meeting the Australian recommendations for alcohol consumption (≤2 standard drinks for men and ≤1standard drink for women) [15].

#### Psychosocial variables

Depression and anxiety were assessed using the Hospital Anxiety and Depression Scale (HADS) [29], a validated instrument used extensively in cardiac populations. It reports high sensitivity to changes both during the course of disease and in response to psychotherapeutic and psychopharmacological intervention [30]. Each item is scored 0–3 with a maximum score of 21 on the depression or anxiety subscales [29]. A HADS score of ≥8 has been identified as an optimal cut-off for case-definition for anxiety disorders and depression based on International Classification of Diseases (ICD-9). We used a continuous variable (mean baseline anxiety and depression scores) and a categorical variable (combined HADS score > or <8) as potential predictors in the analysis. The ENRICHD Social Support Instrument (ESSI) [31] was used to assess self-reported social support. It is a seven-item scale where items are summed to generate a total score, with higher scores indicating greater social support.

### Statistical analyses

Six-month outcomes were described using mean and standard deviation (SD). Initially bivariate linear regression models were used to examine the association between baseline predictors (demographic and clinical, behavioural, and psychosocial variables) and the two outcome variables (physical and mental HRQoL). The results were not significantly different between treatment groups (data not shown), so results for all participants have been presented. A forward stepwise logistic regression model was constructed and the first variable in the model was the one most significantly associated with the outcome. Variables were successively added to the model and retained if they added significant information at the *p*<0.05 level as assessed by the likelihood ratio test. Collinearity between potential predictor variables was examined by calculating their variance inflation factor and those that were highly collinear were eliminated from the final model. Statistical significance was set at *p*≤0.05. Analyses were conducted using Stata statistical software (Stata Corporation, College Station, Texas, United States).

## Results

We recruited 430 participants (86% of those who were eligible) and obtained 6-month follow-up data on 337 (78%). There were no significant differences in baseline characteristics between study completers and those who withdrew or were lost to follow up (data not shown). Two participants died of unknown causes, and 2 were highly depressed/suicidal, so were excluded from the study and analysis. Flow of participants through the trial and baseline characteristics have been reported previously [17]. This paper reports data for 294 (68%) participants who had complete data for physical and mental HRQoL at 6 months.

Participants were: mostly male [232 (79%)]; middle aged [mean = 60.5 years (SD = 10.7)]; married [205 (70%)]; had completed at least high school [242 (82%)], were employed [140 (48%)]; had an annual income over AUD$65,000 [63 (24%)]; had received coronary interventions post-MI [254 (63%) including 153 (55%) who had percutaneous coronary intervention and 27 (10%) who had coronary bypass surgery]; had a family history of CHD [192 (68%)]; had diabetes [68 (23%)]; had hypertension [162 (55%)]; and had participated in other cardiac rehabilitation programs [83 (28%)]. The sample were also characterised by poor lifestyle factors at baseline as the majority were overweight/obese [69% or n =184; Mean BMI = 28.2, SD = 5.68)]; 33% (n = 96) were current smokers, and only 33% (n = 98) were sufficiently active. Finally, 215 participants (50%) were randomised to receive the ProActive Heart intervention and 203 (94.4%) completed the 6 month intervention.

### Outcome variables

Baseline and 6 month physical HRQoL scores [mean (SD)] were 35.5 (10.0) and 45.5 (11.2) respectively and the mean improvement (95% CI) at 6 months compared with baseline was 10.0 (8.7, 11.3); p<0.001. Baseline and 6 month mental HRQoL scores [mean (SD)] were 46.1 (12.3) and 50.5 (11.1) respectively and the mean improvement (95% CI) at 6 months compared with baseline was 4.5 (3.1, 5.8); *p*<0.001.

### Predictor variables

Bivariate associations between predictor variables and physical and mental HRQoL at 6 months are presented in Table 1 (demographic and clinical variables), Table 2 (health behavioural variables), and Table 3 (psychosocial variables).

**Table 1:** Associations between baseline demographic/clinical variables and six month health-related quality of life (HRQoL) outcomes (N=294)

		**Physical HRQoL**			**Mental HRQoL**		
**Characteristic**	**N**	**Mean (SD)**	**Mean diff**	***p***	**Mean (SD)**	**Mean diff**	***p***
			**(95% CI)**			**(95% CI)**	
Age							
Age ≤60	141	47.7 (10.2)			48.5 (11.3)		
Age >60	153	43.5 (11.8)	-4.2 (-6.8, -1.7)	0.001	52.4 (10.7)	3.9 (1.4, 6.4)	<0.01
Gender							
Female	62	44.1 (11.8)			48.3 (12.7)		
Male	232	45.9 (11.1)	1.8 (-1.4, 5.0)	0.27	51.1 (10.6)	2.8 (-0.3, 5.9)	0.08
Marital Status							
Not married	87	44.3 (11.6)			50.5 (11.9)		
Married/de-facto relationship	207	46.0 (11.1)	1.8 (-1.1, 4.6)	0.22	50.62 (10.8)	0.1 (-2.7, 2.9)	0.92
Education							
Did not complete high school	52	42.2 (10.8)			49.6 (14.0)		
Completed at least high school	242	46.2 (11.2)	4.0 (0.7, 7.4)	0.02	50.7 (10.4)	1.1 (-2.2, 4.5)	0.51
Employment							
Unemployed/Retired	154	41.2 (12.1)			48.4 (12.2)		
Employed	140	48.8 (9.5)	7.6 (3.8, 11.4)	<0.001	50.1 (10.0)	1.7 (-2.1, 5.5)	0.39
Income							
Income <AUD$65,000/annum^a^	196	44.0 (12.2)			50.9 (11.0)		
Income ≥AUD$65,000/annum^a^	63	50.2 (7.5)	6.2 (3.0, 9.4)	<0.001	49.2 (11.4)	-1.7 (-4.9, 1.4)	0.28
Medical Procedure^b^							
Non-invasive procedure (e.g. angiogram)	98	44.3 (11.7)			51.1 (11.5)		
Percutaneous coronary intervention	153	45.8 (11.2)	1.5 (-1.4, 4.4)		50.8 (10.5)	-0.3 (-3.2, 2.6)	
Coronary bypass surgery	27	46.9 (10.6)	2.6 (-2.3, 7.5)	0.29	48.4 (14.0)	-2.7 (-7.5, 2.1)	0.27
Participation in another cardiac rehabilitation program							
No	210	46.1 (9.6)			49.3 (12.4)		
Yes	84	44.5 (10.9)	1.3 (-1.6, 4.1)	0.39	50 5 (10.1)	-1.6 (-4.5, 1.2)	0.26
Family History of Heart Disease^c^							
No	91	47.0 (10.0)			52.3 (10.1)		
Yes	192	44.9 (11.7)	-2.2 (-0.6, 5.0)	0.12	50.0 (11.5)	-2.3 (-0.5, 5.1)	0.10
Diabetes							
No	226	46.6 (10.4)			51.0 (10.4)		
Yes	68	41.9 (13.0)	-4.7 (-1.6, -7.7)	<0.01	49.0 (13.2)	-2.0 (-5.1, 1.0)	0.18
Hypertension							
No	132	47.4 (10.3)			51.0 (10.2)		
Yes	162	44.0 (11.8)	-3.3 (-5.9, -0.8)	0.01	50.1 (11.9)	-0.9 (-3.4, 1.7)	0.51
Body Mass Index, kg/m^2 d^							
Healthy weight	81	47.5 (10.0)			49.0 (11.1)		
Overweight	100	46.0 (11.5)	-1.5(-4.8, 1.8)		52.1 (10.0)	3.1 (-0.2, 6.4)	
Obese	84	43.3 (12.0)	-4.2(-7.6, -0.8)	0.02	48.6 (12.5)	-0.4 (-3.8, 3.1)	0.83
Waist circumference, cm^e^							
Healthy waist (<80 cm: women, <94 cm: men)	85	47.2 (10.1)			50.3 (10.4)		
Increased risk (≥80 cm: women, ≥94 cm: men)	189	44.9 (11.6)	-2.3(-5.2, 0.6)	0.12	50.7 (11.4)	0.3 (-2.5, 3.2)	0.82
Randomisation to group							
Intervention	141	44.8 (11.8)			51.8 (9.8)		
Usual care	153	46.1 (10.7)	-1.3 (-3.9, 1.3)	0.33	49.3 (12.1)	2.5 (-0.0, 5.1)	0.04

Due to missing data: ^a^N=259, ^b^N=278, ^c^N=283, ^d^N=265, ^e^N=274.

**Table 2:** Associations between baseline behavioural variables and six month health-related quality of life (HRQoL) outcomes (N=294)

		**Physical HRQoL**			**Mental HRQoL**		
**Characteristic**	**N**	**Mean (SD)**	**Mean diff**	***p***	**Mean (SD)**	**Mean diff**	***p***
			**(95% CI)**			**(95% CI)**	
Physical Activity							
Insufficient (<150 minutes/week)	196	44.8 (11.6)			50.3 (11.4)		
Sufficient (≥150 minutes/week)	98	46.9 (10.3)	2.0 (-0.7, 4.8)	0.15	50.9 (10.6)	0.6 (-2.1, 3.3)	0.65
Intention to be physically active^a^							
No intention to be physically active in next 6 mths	16	30.0 (13.4)			47.5 (15.6)		
Intend to be physically active in next 6 mths	277	46.5 (10.4)	16.4 (11.1, 21.8)	<0.001	50.6 (10.8)	3.1 (-2.5, 8.7)	0.28
Physical activity self-efficacy^a^	293	-	2.9 (2.2, 3.6)	<0.001	-	1.1 (0.3, 1.8)	<0.01
Television viewing, hours/week^b^	289	-	-0.3 (-0.4, -0.1)	<0.001	-	-0.1 (-0.2, 0.1)	0.08
Vegetable intake^a^							
<5 serves/week	221	45.2 (11.0)			50.3 (10.9)		
≥5 serves/week	72	46.6 (11.8)	1.4 (-1.6, 4.4)	0.37	60.0 (11.8)	0.7 (-2.3, 3.6)	0.63
Fruit intake^a^							
<2 serves/week	158	46.5 (10.9)			50.7 (11.3)		
≥2 serves/week	135	44.5 (11.5)	-2.0 (-4.5, 0.6)	0.14	50.2 (10.9)	-0.5 (-3.1, 2.1)	0.70
Total fat intake							
>30% total energy intake/day	243	44.9 (11.5)			50.3 (11.7)		
≤30% total energy intake/day	51	48.3 (9.3)	3.3 (-0.1, 6.7)	0.05	51.8 (7.8)	1.5 (-1.9, 4.9)	0.38
Sodium intake^a^							
≥2300 mg/day	65	44.7 (11.5)			49.4 (11.4)		
<2300 mg/day	228	45.7 (11.2)	1.0 (-2.1, 4.1)	0.52	50.8 (11.1)	1.4 (-1.7, 4.5)	0.37
Cholesterol intake							
>300 mg/day	86	45.8 (10.2)			49.9 (11.3)		
≤300 mg/day	208	45.3 (11.7)	-0.5 (-3.3, 2.4)	0.75	50.8 (11.1)	0.9 (-2.0, 3.7)	0.55
Alcohol intake^a^							
>2 std drink/day (male) />1std drink/ day (female)	89	46.8 (9.3)			51.0 (10.4)		
≤2std drink/day (male) /≤1std drink/ day (female)	204	45.0 (12.0)	-1.8 (-4.6, 1.0)	0.22	50.2 (11.4)	-0.8 (-3.6, 2.0)	0.57
Ever smoked							
No	75	45.2 (11.0)			50.9 (12.3)		
Yes	219	45.6 (11.3)	0.4 (-2.5, 3.4)	0.77	50.4 (10.7)	-0.5 (-3.4, 2.4)	0.74

Due to missing data: ^a^N=293, ^b^N=289.

**Table 3:** Associations between baseline health-related quality of life (HRQoL) and psychosocial variables, and 6 month HRQoL (N=294)

			**Physical HRQoL**			**Mental HRQoL**	
**Characteristic**	**N**	**Mean (SD)**	**Mean difference**	***p***	**Mean (SD)**	**Mean difference**	***p***
			**(95% CI)**			**(95% CI)**	
HRQoL (0–100)^a^	294						
Physical HRQoL		-	0.5 (0.4, 0.6)	<0.001	-	0.1 (0.0, 0.3)	0.04
Mental HRQoL		-	0.2 (0.1, 0.3)	<0.001	-	0.4 (0.4, 0.5)	<0.001
Physical Functioning (PF)		-	0.4 (0.3, 0.5)	<0.001	-	0.2 (0.1, 0.3)	<0.001
Role Physical (RP)		-	0.3 (0.2, 0.4)	<0.001	-	0.2 (0.0, 0.3)	0.01
Bodily Pain (BP)		-	0.2 (0.1, 0.3)	<0.001	-	0.2 (0.1, 0.2)	<0.01
General Health (GH)		-	0.7 (0.6, 0.8)	<0.001	-	0.3 (0.2, 0.5)	<0.001
Vitality (VT)		-	0.4 (0.3, 0.5)	<0.001	-	0.4 (0.3, 0.5)	<0.001
Social Functioning (SF)		-	0.2 (0.1, 0.3)	<0.001	-	0.3 (0.2, 0.4)	<0.001
Role Emotional (RE)		-	0.2 (0.1, 0.3)	<0.001	-	0.3 (0.2, 0.3)	<0.001
Mental Health (MH)		-	0.3 (0.2, 0.4)	<0.001	-	0.5 (0.4, 0.6)	<0.001
Anxious^b^ (range 0–21)							
No (0–7)	205	46.7 (10.9)			53.6 (8.7)		
Yes (8–21)	88	42.8 (11.5)	-4.0 (-6.8, -1.2)	<0.01	43.3 (12.9)	-10.3 (-12.8, -7.7)	<0.001
Depressed^b^ (range 0–21)							
No (0–7)	241	46.7 (10.9)			53.0 (8.5)		
Yes (8–21)	52	40.2 (11.5)	-6.6 (-9.9, -3.3)	<0.001	39.2 (14.5)	-13.8 (-16.7, -10.8)	<0.001
Social Support^a,b^ (range 10–34)	293	-	0.4 (0.2, 0.7)	<0.001	-	0.6 (0.4, 0.9)	<0.001

^a^Higher scores indicate greater functioning. ^b^Due to missing data N=293.

When multiple regression analysis was performed, older age, unemployment, lower baseline physical and mental HRQoL, lower confidence levels in meeting sufficient physical activity guidelines, no intention to be physically active in the next 6 months, and greater sedentary behaviour were strong independent predictors of lower physical HRQoL at 6 months. The model explained 43% of the variance. While younger age, lower baseline mental HRQoL, depression, lower social support, and greater sedentary behaviour were predictors of lower mental HRQoL at 6 months. The model explained 37% of the variance (Table 4).

**Table 4:** Significant baseline predictor variables of 6 month health-related quality of life (HRQoL) from multivariate model (N=294)

	**Physical HRQoL**		**Mental HRQoL**	
**Characteristic**	**Mean difference**	***p***	**Mean difference**	***p***
	**(95% CI)**		**(95% CI)**	
Age				
Age ≤60				
Age >60	-4.9 (-7.6, -2.2)	<0.001	2.8 (0.7, 4.9)	0.01
Employed				
Unemployed/Retired				
Employed	3.3 (0.2, 6.4)	0.03	-	-
Baseline Physical HRQoL	0.4 (0.2, 0.5)	<0.001	-	-
Baseline Mental HRQoL	0.2 (0.1, 0.3)	<0.001	0.3 (0.2, 0.4)	<0.001
Physical activity self-efficacy	1.4 (0.7, 2.1)	<0.001	-	-
Intention to be physically active				
No intention to be physically active in next 6 mths				
Intend to be physically active in next 6 mths	9.4 (4.8, 14.1)	<0.001	-	-
Television viewing	-0.2 (-0.3, -0.1)	0.001	-0.1 (-0.2, -0.0)	0.01
Depressed (range 0–21)				
No (0–7)				
Yes (8–21)	-	-	-7.4 (-10.6, -4.1)	<0.001
Social Support^a^ (range 10–34)	-	-	0.4 (0.2, 0.6)	0.001

^a^Higher scores indicate greater social support.

## Discussion

This study identified independent baseline predictors of physical and mental HRQoL six months after hospitalisation for MI. As reported here, baseline HRQoL scores have been shown to be significant predictors of subsequent physical and mental HRQoL outcomes for various populations of cardiac patients [14,32,33]. Importantly, low HRQoL impacts the recovery process, decreases compliance with treatments, decreases capacity to perform activities of daily living, increases the rate of hospital admission, and puts the patient at risk for complications and death [34,35].

Previous investigators have shown that sociodemographics are significant predictors of HRQoL. Consistent with our findings, recent studies found that younger age was significantly associated with higher physical HRQoL and older age was associated with higher mental HRQoL [36,37]. Beck et al. [8] suggest that treatment differences between younger and older MI patients may account for the association between age and HRQoL, as younger patients are treated more aggressively. Also, gender-related differences in HRQoL have been reported among coronary patients with women not coping as well physically and psychosocially as men, although the literature is inconsistent and it remains unclear why these differences exist [11,29,38]. Therefore, further research is required to fully investigate the association between age, gender and HRQoL for MI patients.

We investigated a range of available clinical characteristics including medical procedure, participation in another cardiac rehabilitation program, family history of heart disease, comorbidities (diabetes and hypertension), BMI and waist circumference but they did not appear to strongly affect patients’ HRQoL. Previous investigators have shown that angina, physical functioning and fatigue have been significant predictors of HRQoL [37], whilst, consistent with our findings, others have found that clinical characteristics (history of heart disease, participation in cardiac rehabilitation, revascularisation procedure) were unlikely to be strong predictors of HRQoL after MI [8]. It is important to note that our study population represents a group of patients who were willing to participate in a research study, so it’s possible that clinical characteristics may be predictors of HRQoL for older patients with more comorbid diseases who may be less likely to participate in a clinical trial [8].

We have previously reported that participants had normal anxiety and depression scores at baseline [39]. However, consistent with the findings of previous investigators [40,41], this study highlighted the negative impact of psychosocial characteristics (e.g. depression, anxiety or social isolation) on HRQoL after MI. Importantly, psychosocial factors are also important predictors of clinical outcomes such as mortality after MI [42-44]. In particular, depression is known to predict outcomes in MI patients, including mortality, health service use and secondary prevention activities such as smoking cessation and medication adherence [42,45-47]. Depression is also related to other psychosocial outcomes such as returning to work after cardiovascular disease, and is associated with failing to increase physical activity [48-50]. These results provide further evidence for the importance of incorporating assessments of psychosocial factors into the initial treatment regimen.

To our knowledge, this is the first study that has reported a range of health behavioural predictors of HRQoL for MI patients. Lower confidence levels in meeting sufficient physical activity guidelines, no intention to be physically active and greater sedentary behaviour were predictors of lower physical HRQoL at 6 months. Whilst less sedentary behaviour was also a predictor of lower mental HRQoL at 6 months. These results highlight the impact of health behaviours on HRQoL, particularly physical activity self-efficacy and intentions, and emphasise the importance of sedentary behaviour. There is emerging evidence suggesting that sedentary behaviour has deleterious health consequences that are distinct from the beneficial effects of moderate-to-vigorous-intensity physical activity [51], as sedentary behaviour is thought to be independently associated with chronic disease-related risk factors such as central adiposity, elevated blood glucose and insulin, and other cardiometabolic biomarkers [52].

Study participants’ physical and mental HRQoL scores were below normal levels for the general population [53,54] and MI populations from other countries [22,55]. The US Medical Outcomes Study reported that MI patients diagnosed within the previous year had a mean (SD) physical HRQoL score of 42.7 (10.0) and mental HRQoL score of 51.7 (8.2) [22] and similar results have been reported by others [55]. Participants in the current study had a lower physical HRQoL score of 35.3 (10.0) and mental HRQoL score of 46.1 (12.3). This difference may be attributable to sociodemographic or clinical differences between the study populations.

Our study strengths include: the well-defined and representative sample of CHD patients with the recruitment of MI patients; the high consent rate; the comprehensive assessment of demographic/clinical, health behavioural and psychosocial predictor variables; the measurement of HRQoL with a widely used, valid and reliable instrument; and the limited loss to follow up for a six month intervention trial. Importantly, patients who participated in other cardiac rehabilitation programs were not excluded from this ‘real-world’ trial. Study limitations include the use of self-reported data that may have been limited by recall error and social desirability, and the use of telephone interview to collect data which limited our ability to collect objective biomedical data. However, the study outcomes were consistent with those reported in previous trials and all measures have been routinely used in population-based epidemiological and intervention research [23,28].

## Conclusions

The study results suggest that assessment of HRQoL, demographics (age, employment), health behaviours (physical activity and sedentary behaviour), and psychosocial functioning (depression and social support) during hospitalisation for MI may be helpful in predicting those who will have impaired physical and mental HRQoL six months later. This would allow for targeted counselling or secondary prevention efforts. These results also highlight the need to implement treatment strategies that have a significant impact on HRQoL as we found that the study sample’s HRQoL scores were below normative levels for the target population.

## Abbreviations

AUD: Australian Dollar; BMI: Body mass index; CHD: Coronary heart disease; ESSI: ENRICHD social support inventory; HADS: Hospital anxiety and depression scale; HRQoL: Health-related quality of life; SD: Standard deviation; SF-36: Short-Form 36 Questionnaire; TV: Television.

## Competing interests

The authors declare that they have no competing interests.

## Authors’ contributions

ALH and BFO developed the study concept and aims, and implemented the study protocol. All authors contributed to the analysis and interpretation of data. ALH drafted the manuscript and all authors contributed to the final version. All authors read and approved the final manuscript.

## Pre-publication history

The pre-publication history for this paper can be accessed here:

http://www.biomedcentral.com/1471-2261/13/69/prepub
